# Measuring the Strength of Interaction between the Ebola Fusion Peptide and Lipid Rafts: Implications for Membrane Fusion and Virus Infection

**DOI:** 10.1371/journal.pone.0015756

**Published:** 2011-01-13

**Authors:** Mônica S. Freitas, Cristian Follmer, Lilian T. Costa, Cecília Vilani, M. Lucia Bianconi, Carlos Alberto Achete, Jerson L. Silva

**Affiliations:** 1 Centro Nacional de Ressonância Magnética Nuclear Jiri Jonas, Instituto de Bioquímica Médica, Instituto Nacional de Ciências e Tecnologia de Biologia Estrutural e Bioimagem, Universidade Federal do Rio de Janeiro, Rio de Janeiro, Brazil; 2 Departamento de Físico-Química, Instituto de Química, Universidade Federal do Rio de Janeiro, Rio de Janeiro, Brazil; 3 Divisão de Metrologia de Materiais (DIMAT), Inmetro, Rio de Janeiro, Brazil; 4 Laboratorio de Biocalorimetria, Instituto de Bioqumica Medica, Universidade Federal do Rio de Janeiro, Rio de Janeiro, Brazil; 5 Programa de Engenharia Metalúrgica e de Materiais, COPPE, Universidade Federal do Rio de Janeiro (UFRJ), Rio de Janeiro, Brazil; University of South Florida College of Medicine, United States of America

## Abstract

The Ebola fusion peptide (EBO_16_) is a hydrophobic domain that belongs to the GP2 membrane fusion protein of the Ebola virus. It adopts a helical structure in the presence of mimetic membranes that is stabilized by the presence of an aromatic-aromatic interaction established by Trp8 and Phe12. In spite of its infectious cycle becoming better understood recently, several steps still remain unclear, a lacuna that makes it difficult to develop strategies to block infection. In order to gain insight into the mechanism of membrane fusion, we probed the structure, function and energetics of EBO_16_ and its mutant W8A, in the absence or presence of different lipid membranes, including isolated domain-resistant membranes (DRM), a good experimental model for lipid rafts. The depletion of cholesterol from living mammalian cells reduced the ability of EBO_16_ to induce lipid mixing. On the other hand, EBO_16_ was structurally sensitive to interaction with lipid rafts (DRMs), but the same was not observed for W8A mutant. In agreement with these data, W8A showed a poor ability to promote membrane aggregation in comparison to EBO_16_. Single molecule AFM experiments showed a high affinity force pattern for the interaction of EBO_16_ and DRM, which seems to be a complex energetic event as observed by the calorimetric profile. Our study is the first to show a strong correlation between the initial step of Ebola virus infection and cholesterol, thus providing a rationale for Ebola virus proteins being co-localized with lipid-raft domains. In all, the results show how small fusion peptide sequences have evolved to adopt highly specific and strong interactions with membrane domains. Such features suggest these processes are excellent targets for therapeutic and vaccine approaches to viral diseases.

## Introduction

The *Filoviridae* family contains the Ebola and Marburg viruses. These are enveloped viruses composed of seven genes which encode eight proteins in the Ebola virus and seven in the Marburg virus [1]. The single-stranded negative-sense RNA genome is encased in a nucleocapsid complex, which consists of the following four viral proteins: the nucleoprotein (NP), the viral proteins (VP35 and VP30) and the polymerase (L). This complex is surrounded by a matrix consisting of VP40 and VP24, which is packaged by a lipid membrane envelope obtained during budding from the host cell. The envelope is composed of the GP protein, which is post-translationally cleaved by a furin protease into two fragments, GP1 and GP2, although this cleavage is not necessary for *in vitro* viral infection of cells [2], [3]. A disulfide bridge in the mature molecule connects these subunits. GP1 is responsible for interaction with its cellular receptor, and GP2 is involved in the mechanism of membrane fusion [4], [5].

Membrane fusion is a common feature among enveloped viruses and is an important part of the viral infection cycle [6]. However, this process can be triggered in different ways. Viruses can enter cells by direct fusion with the cell plasma membrane or through the endocytic pathway [7], [8]. Fusion is mediated by the viral envelope protein that contains a nonpolar fusion peptide. In general, fusion peptides that belong to class I viral fusion proteins are located at the N-terminus, whereas in class II, they are in the internal region [9]. However, in both cases they are typically rich in alanine and glycine residues and highly conserved within a virus family [10]. The interaction of the fusion peptide with target membranes is critical for fusion. Therefore, this region has to be exposed at the proper place and time in order to trigger the interaction.

The Ebola fusion peptide is a highly conserved hydrophobic sequence of about 16 amino acids (^524^GAAIGLAWIPYFGPAA^539^) [11]. Recently, we have solved the NMR atomic structure of the Ebola fusion peptide in the presence of mimetic membranes, where a loop with a central 3_10_-helix appears to be stabilized by aromatic-aromatic interaction [12]. The ability of the Ebola peptide to induce membrane fusion has been related with the presence of phosphatidilinositol in the host cell membrane and Ca^2+^ during this process [13], [14].

Recent studies have suggested the critical role of lipid rafts in filovirus entry into the host cells. Lipid rafts are microdomains in biological membranes that are rich in cholesterol and sphingolipids and play an important role in many events including the endocytic, bio-synthetic and signal transduction pathways [15], [16], [17]. The requirement of lipid rafts for the virus to enter host cells has been related with the localization of receptors and co-receptors in these microdomains [18], [19]. Many viruses use a specific interaction between their GPs and cell surface receptors to initiate the attachment to cells and subsequent fusion. Thus, lipid rafts may promote virus entry by concentrating the viral receptors and facilitating binding via an efficient interaction of these receptors with viral proteins. Interestingly, the filovirus co-factor folate receptor-α (FRα) is a raft-associated glycophosphatidylinositol-anchored protein [20], [21]. However, the critical role of FRα has been questioned due to the fact that FRα ~negative cells are fully infectible by GP pseudotypes [22].

In order to determine the importance of cholesterol during membrane fusion and the real importance of the aromatic-aromatic interaction in the peptide structure, we studied the interaction of the wild type (wt) fusion peptide and its mutant W8A peptide with either cholesterol-depleted cells or rafts isolated from Vero and BHK-21 cells. Our results show that the Ebola fusion peptide interacts with living cells, and its capacity to induce cell-cell fusion is decreased in cholesterol-depleted cells. Force spectroscopy based on atomic force microscopy (AFM) assays reveals a pattern of high affinity force when the Ebola fusion peptide interacts with membrane rafts. It is also observed that the peptide is able to induce aggregation of the lipid rafts, suggesting an important role for phosphatidylinositol and cholesterol during entry of the virus into the target cells.

## Results

### Cholesterol depletion and cell viability

The lipid composition and the curvature of biological membranes are limiting steps for peptide interactions with living cells and liposomes [23], [24]. Cholesterol has been proven to be essential for filovirus replication, and the entry of the Ebola and Marburg viruses is inhibited after cholesterol depletion of the target cells [25]. In cells not depleted of cholesterol, viral proteins co-localize with caveolin after internalization [25]. Caveolae are vesicles enriched with cholesterol and sphingolipids and have been shown to be involved in a wide range of biological events such as cellular entry by certain viruses [26], [27].

**Figure 1:**
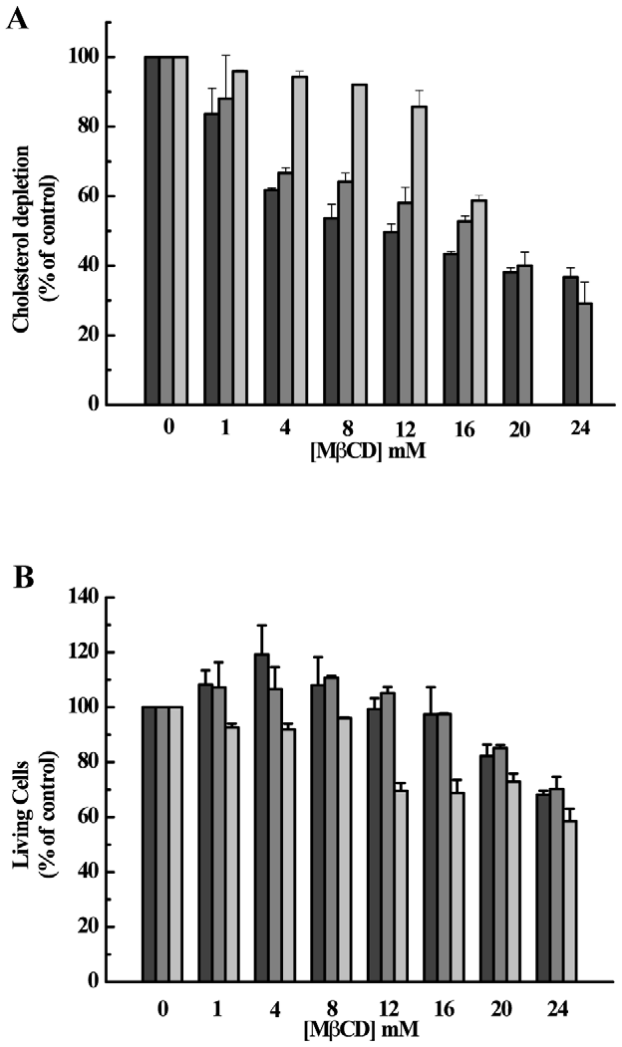
Effect of MβCD on cell viability. (A) Cholesterol depletion. Cells were pre-treated with MβCD for 30 min at 37°C, and cholesterol content was quantified as described in Experimental Procedures. (B) Cell viability. Cells were incubated with MβCD for 30 min at 37°C. Then MTT reagent was added and incubated for 4 h. The cells were incubated overnight with the solubilization buffer. Absorbance was measured at 570 nm. The bars represent BHK-21, VERO and C636, respectively from left to right.

In this work, we depleted cholesterol from cells to understand its importance in the mechanism of membrane fusion, an early step in the Ebola infection cycle. Since Vero and BHK-21 mammalian cells are permissive to infection mediated by the Ebola virus, initial attempts were performed by using those cells [28]. β-cyclodextrins were used to since they are very effective to selectively extract cholesterol from membranes of intact cells without binding or insertion into the plasma membrane [29], [30]. Vero and BHK-21 cells were treated with increasing concentrations of MβCD for 30 min at 37°C and then assayed for cholesterol quantification. As shown in Fig. 1A, cholesterol depletion was dose-dependent for Vero and BHK-21 cells. In addition, insect cells (C6/36), previously grown in medium with cholesterol, were assayed as a cellular control of low cholesterol content cells. Insect cells are cholesterol auxotrophs and can be depleted of cholesterol by growth in delipidated serum. As observed in Fig. 1A, the cholesterol content of C6/36 cells was maintained after incubation with up to 12 mM MβCD. However, upon incubation with 20 and 24 mM MβCD, it was not possible to detect cholesterol due to the low cell adhesion induced by depletion. To determine the effect of MβCD on cell viability, Vero, BHK-21 and C6/36 cells were incubated in the absence or in the presence of MβCD. At the same time we added MTT reagents to prevent cellular loss during the washing step (see Experimental Procedures). In general, insect and mammalian cell monolayers were intact after 30 min incubation with up to 16 or 24 mM of MβCD, respectively (data not shown). Fig. 1B shows that insect cells were more affected by cholesterol depletion than mammalian cells. Indeed, 16 mM MβCD was able to decrease 50–60% of cholesterol in mammalian and insect cells but only affected the viability of insect cells (Fig. 1B). Thus, our results showed that some different MβCD concentrations can induce similar levels of cholesterol depletion but different responses in cellular viability. In our studies, low MβCD concentrations, which cause depletion of cholesterol but do not affect the cellular viability, were chosen to examine the role played by cholesterol during protein-membrane interaction.

### Ebola fusion peptide and lipid membrane interaction

**Figure 2:**
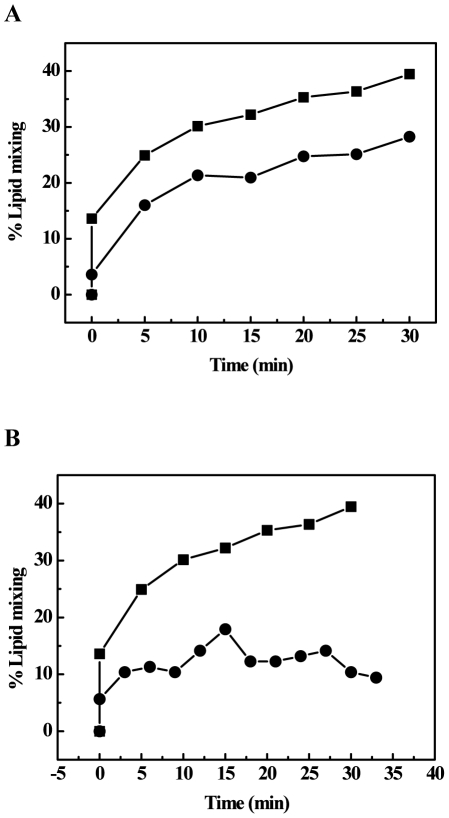
Peptide membrane interaction. (A) Living Vero cells were incubated with wtEBO_16_ at 25 (circles) or 37°C (square). Membrane mixing was followed by the decrease in the pyrene excimer/monomer fluorescence ratio for 30 min. (B) Living Vero cells in the absence (square) or in the presence (circle) of MβCD were incubated with wtEBO_16_ at 37°C. Membrane mixing was followed by the decrease in the pyrene excimer/monomer ratio for 30 min. The peptide concentration was 100 µM in all experiments. The MβCD concentration was 20 mM. The percentage of lipid mixing was obtained by the relation described in Freitas et al [12].

To study the ability of the wild type Ebola fusion peptide (wtEBO_16_) to induce cell-cell fusion, we synthesized a small hydrophobic peptide, previously described as a region belonging to the GP2 protein that interacts with target cells during virus-cell fusion [11], [31]. Vero cells were incubated at 25 or 37°C, and fusion reactions were started by the addition of wtEBO_16_. Our data showed that the Ebola fusion peptide was able to induce cell-cell fusion at neutral pH and in the absence of Ca^2+^ (Fig. 2A). As expected, despite the fact that the fusion process occurred at both temperatures, it was slower at 25°C. In order to determine the importance of cholesterol in the fusion process, Vero cells were depleted of cholesterol by pre-treatment with MβCD. As shown in Fig. 2B, cholesterol-depleted cells were less susceptible to lipid mixing, indicating that cholesterol is important for fusion.

Previous studies had shown that low endosomal pH is required for infection and cell-cell fusion mediated by Ebola virus GP [32], [33] and that low pH is required for optimal functioning of cathepsin B and L, which are important to the initial step of Ebola virus entry into target cells [34]. However, there is no information concerning any association between low pH and membrane fusion. As EBO_16_ does not have any amino acid with pKa lower than 7, it should not be expected any effect under lower pH.

### Secondary structure induced by membrane interaction

**Figure 3:**
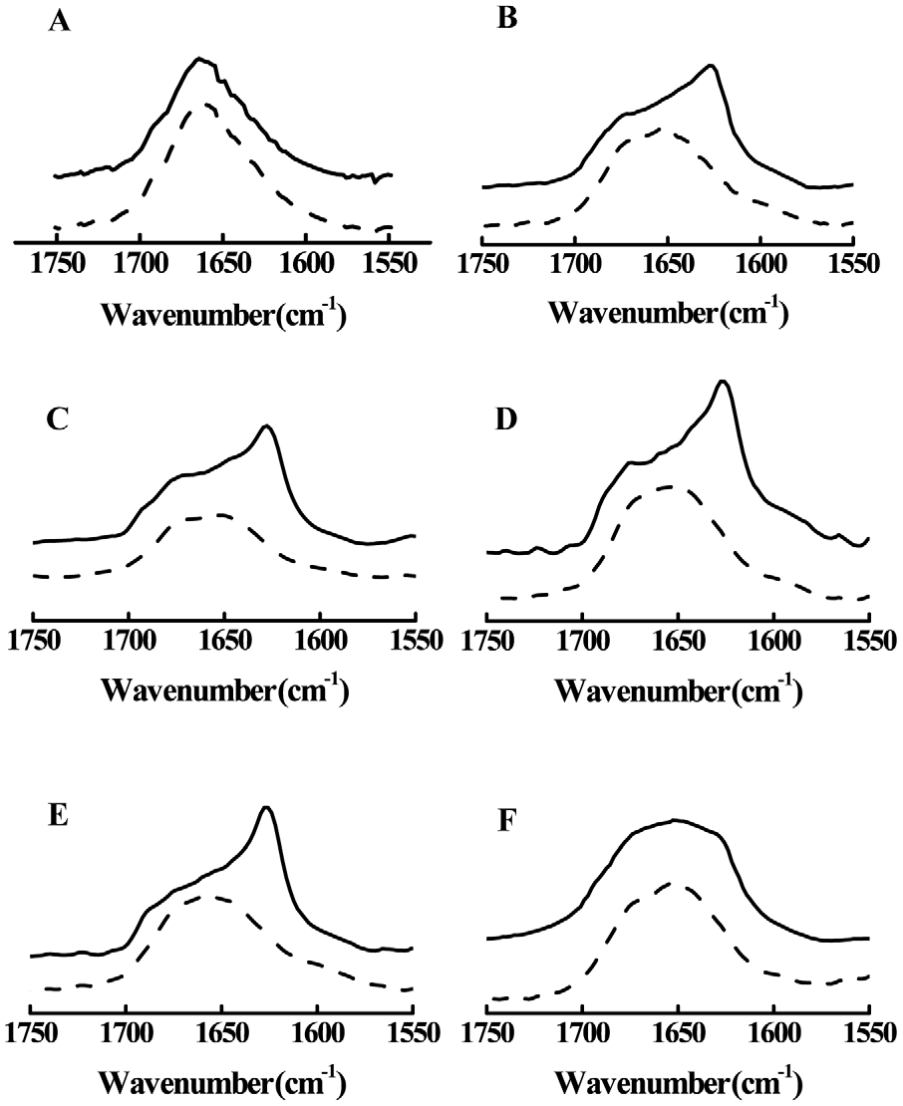
Effect of lipid vesicles in the FTIR spectra of the amide I band of wtBO_16_ (solid lines) and W8A (dashed lines) at 25°C. Peptides in 100% (A) and 50% (B) DMSO or in the presence of LUVs of different compositions: PC (C), PC∶PE∶PI∶Cho (D), PC∶PE∶SPM∶Cho (E), or in the presence of lipid rafts from Vero cells (F). The peptide concentration was 19.5 mM, and the LUV concentration was 44 mM. The ordinate represents absorption (in arbitrary units).

Secondary structures of wt and its mutant W8A in the presence of vesicles were examined using conventional FT-IR spectroscopy. Representative spectra of the amide I band for the peptides in the absence or in the presence of vesicles are shown in Fig. 3. The amide I band consists of the C = O stretching (76%), C-N stretching (14%) and C-C-N deformation (10%) modes and appears in the region from 1600 to 1700 cm^−1^. This band is highly sensitive to the secondary structure of proteins and serves as an indicator of α-helix, β-sheet, turn and random conformation. Both the wt and W8A peptides diluted in DMSO showed a similar profile, with broad spectra at a maximum around 1665 cm^−1^ (Fig. 3A). In general, peaks in between 1680 and 1660 are related to turn, suggesting an unfolded structure in the presence of a high amount of DMSO. However, in the presence of 50% DMSO, it was possible to observe a peak that arose at approximately 1625 cm^−1^, suggesting an increase of b-sheet structure for wtEBO_16_ that was not observed for W8A (Fig. 3B). The increase in β-sheet structure could be linked to peptide aggregation induced by the contact with water, since a flared spectrum, as observed for W8A, could be correlated to an increase of other structural components.

As previously described by Suarez et al. (2003), the structure of the Ebola fusion peptide can be correlated to the ability of the peptide to perturb membranes, either by increasing permeability or leading to fusion [14]. Thus, we prepared vesicles with different lipid compositions to probe the role that some lipids play during membrane recognition and compared the results with detergent-resistant membranes (DRMs) extracted from VERO cells. In general, interaction between fusion peptide and lipid membrane does not happen in a promiscuous fashion; rather, it is dependent on membrane composition and curvature. To follow the structural behavior adopted by wt and mutant peptides during membrane interaction, we prepared large unilamellar vesicles (LUVs) and DRMs. In the presence of different lipid compositions the wtEBO_16_ showed a distinct structural profile in comparison to EBO_16_ W8A. The structural components observed for wtEBO_16_ and EBO W8A in the presence of 50% DMSO were present when these peptides were incubated with membranes of different LUV compositions, suggesting a poor structural response in these cases (Fig. 3C, D and E).

On the other hand, the conformational exchange undergone by wtEBO_16_ in the presence of DRMs reinforces the requirement of a specific lipid composition in membranes during binding that drives the wtEBO_16_ aggregation into a folded state. The flared peak observed for both peptides in the presence of DRMs is representative of several mixed structural components, possibly suggesting a non-homogeneous correlation between binding and structure (Fig. 3F). The data suggest a kind of structural fluctuation that could be stabilized by the full extension of the membrane protein (GP2 protein).

### Peptide-membrane raft interaction followed by Single Molecule Force Spectroscopy

**Figure 4:**
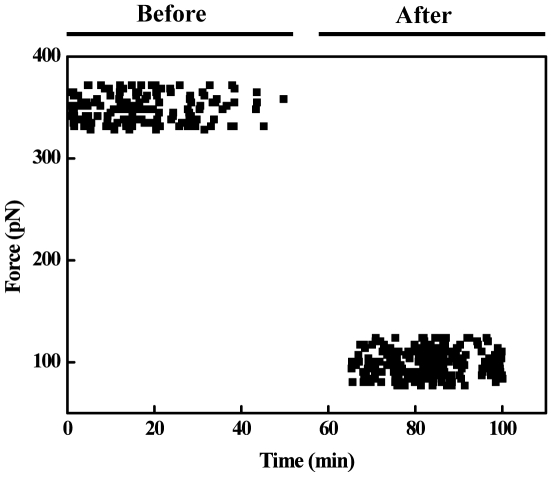
The AFM measurement of the molecular forces associated with EBO_16_-DRM interactions. Force distributions over time before and after the system was saturated with 1 mM wtEBO_16_. All experiments were done in phosphate buffer at room temperature.

To investigate how specific the interaction between the Ebola fusion peptide and microdomains is, we have carried out single molecule force spectroscopy assays. We have measured the direct adhesion force between the Ebola fusion peptide and isolated microdomains (DRMs) from VERO cells. DRMs extracted from the cells as small vesicles were applied onto a glass surface, and the peptide was covalently immobilized to the AFM tip (see Experimental Procedures). Several cycles of the force-distance curve were recorded (Fig. 4). The data show a specific interaction between the peptide and the isolated rafts with an interaction force of about 416±25 pN (Fig. 4). As expected for a specific interaction, the force values decreased to 75±25 pN when DRMs membranes were saturated with wtEBO_16_ (1 mM) (Fig. 4).

### Vesicle aggregation induced by Ebola fusion peptide

**Figure 5:**
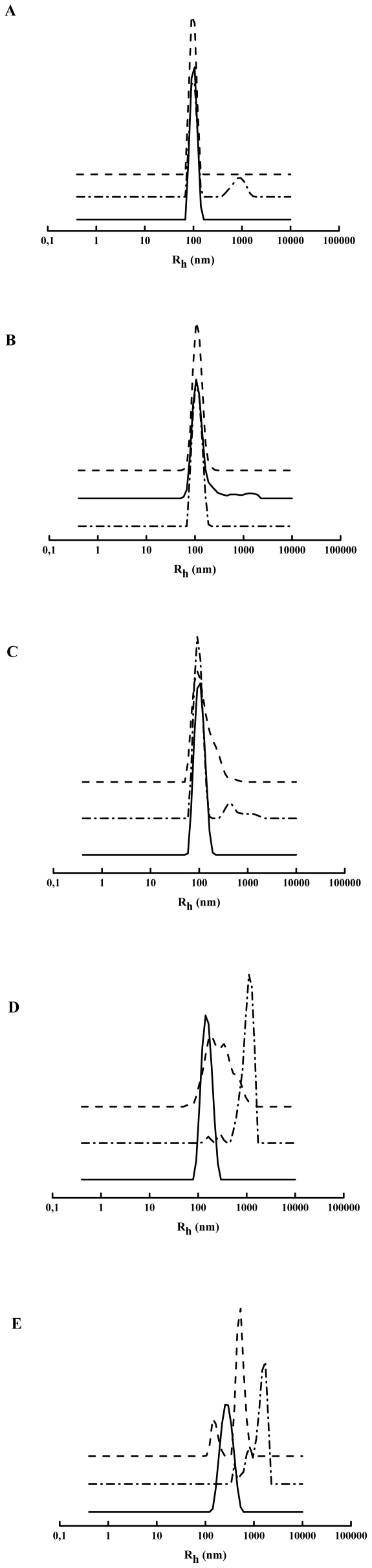
Dynamic light scattering of vesicles incubated with Ebola fusion peptide. (A) PC LUVs, (B) PC∶PE∶PI∶Cho LUVs, (C) PC∶PE∶SPM∶Cho LUVs, (D) lipid rafts from BHK-21 cells and (E) lipid rafts from Vero cells. All experiments were done in phosphate buffer pH 7 at 37°C. The peptide concentration was 100 µM, and the liposome concentration was 1 mM.

Vesicle aggregation induced by Ebola fusion peptide was evaluated by dynamic light scattering (DLS). The incubation of wtEBO_16_ with PC liposomes (apparent hydrodynamic radius of 100 nm) resulted in a new liposome population with an Rh between 800 and 900 nm (Fig. 5A), which represents 23.2% of the total population. In contrast, EBO_16_ W8A was not able to induce PC vesicle aggregation in the same conditions (Fig. 5A). As it can be observed in Fig. 5, the extent of membrane perturbation was dependent on lipid composition. Despite of the fact that polydispersion had shown a similar profile for vesicles composed of PC∶PE∶PI∶Cho and PC∶PE∶SPM∶Cho, the effects on vesicles size were completely distinct (Fig. 5B and 5C). In the presence of PC∶PE∶PI∶Cho (Fig. 5B), the mutant W8A was not able to induce vesicle aggregation, while wtEBO_16_ induced a slight aggregation (Fig. 5B and 5C). In the case of PC∶PE∶SPM∶Cho vesicles (Fig. 5C), both peptides were able to shift a small population of the initial vesicles into an aggregate form, indicating that the binding efficiency was not enough to lead into aggregation, as is also shown by FTIR (Fig. 5C).

On the other hand, under interaction with DRMs, it was possible to see a clear dependence on both membrane composition and peptide structure. As observed in Figs. 5D and 5E, the initial populations of DRM vesicles displayed a more heterogeneous profile than the liposomes. The effects on membrane aggregation induced by both peptides in the presence of DRMs from BHK-21 and VERO cells were different, reflecting a difference in lipid composition. For BHK-21 DRMs, both peptides were able to induce agreggation. However, for EBO_16_ W8A, a small amount of the initial DRM population remained, and a broad range of vesicles sizes, as well as an increased polydispersity, was detected. For VERO DRMs, the initial vesicles were completely converted into aggregates while in the presence of the peptides. However, wtEBO_16_ induced huge aggregates, with a z-average of 1690 nm, while EBO_16_ W8A splitted the original peak into two populations: one with smaller sizes (z-average of 597 nm) and the other with larger sizes. These data reinforce the ability of the Ebola fusion peptide to interact with DRMs and trigger vesicle aggregation, an ability which could be associated with membrane fusion. The wEBO_16_ was more efficient to induce membrane fusion than the EBO_16_W8A suggesting that Trp8 is important for the observed effects.

### Energetic behavior of peptide membrane interaction

**Figure 6:**
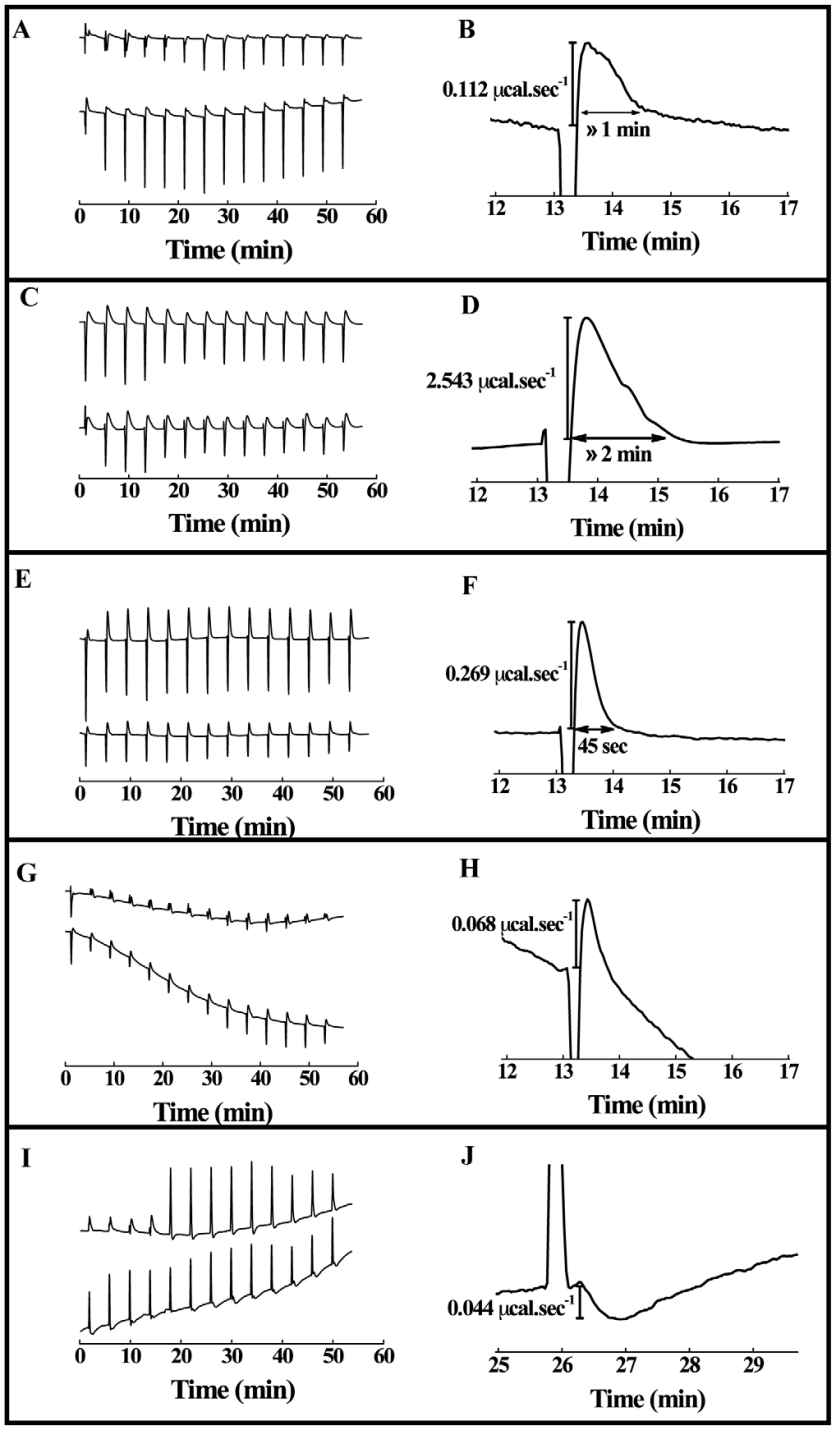
Binding of wtEBO_16_ and W8A to lipid membranes by isothermal titration calorimetry. Each peak corresponds to a 5 µL injection of vesicles into the sample cell containing a 100 µM solution of wtEBO_16_ (a) or W8A (b) peptide. (A) PC LUVs, (C) PC∶PE∶PI∶Cho LUVs (2∶1∶0.5∶1), (E) PC∶PE∶SPM∶Cho LUVs (1∶1∶1∶1.5) LUVs, (G) lipid rafts from BHK-21 cells and (I) lipid rafts from VERO cells. (B), (D), (F), (H) and (J) are enlarged views of selected heat peak of titration experiments shown in (A, b), (C, b), (E, b), (G, b) and (I, b) curves, respectively. All measurements were conducted at 37°C in phosphate buffer pH 7. The peaks were obtained after subtraction of the heat of dilution of the vesicles into buffer from the raw data obtained with the peptides.

To examine the energetic behavior of the peptide membrane interaction, we used calorimetric titration. The heat absorbed or released during the binding reaction reflects the overall energy of peptide-lipid interaction. In the first injections it is expected that all or at least most of the peptide binds to the membranes and the observed hat effect is usually the maximum; after a few injections, the heat effect should decrease because of progressive binding, leading to a saturation of binding sites in the membranes. However, in all isotherms shown here we did not observe a continuous decrease of the absolute value of the heat effect. As shown in Fig. 6A, the injections were followed by two peaks. The first peak reflects the exothermic binding between the peptide and PC liposomes and the second peak represents an endothermic component that could be related to another energetic contributions triggered by peptide-liposome binding, such as membrane destabilization and peptide conformational changes. Although the binding of both peptides was exothermic, the binding of wtEBO_16_ was slightly more exothermic than the binding of W8A mutant for PC liposomes (Fig. 6A, a and b). In addition, the endothermic process was very fast in both cases (≈1 min), and its contribution was greater for wtEBO_16_ than for EBO_16_ W8A peptide (Fig. 6B). Since no modifications of peptide structure was observed while wtEBO_16_ was able to induce PC vesicle aggregation and to promote vesicle leakage (data not shown), the endothermic behavior was correlated with these events, since modification of peptide structure was not observed. Similar titrations were performed in the presence of PC∶PE∶PI∶Cho and PC∶PE∶SPM∶Cho vesicles (Fig. 6E and G). In the case of the interaction between wtEBO_16_ or the mutant W8A and PC∶PE∶PI∶Cho vesicles, a similar exothermic binding contribution was observed (Fig. 6C, a and b). The event correlated to the endothermic peak was slower (≈2 min) than that observed in the presence of PC liposomes (Fig. 6D). On the other hand, for PC∶PE∶SPM∶Cho, the binding of wtEBO_16_ was less exothermic than the binding of EBO_16_-W8A (Fig. 6E, a and b), and the endothermic peak was sharper and the event correlated to it was faster (≈45 sec) (Fig. 6F). Thus, the data show that wtEBO_16_ and its mutant EBO_16_-W8A can interact with membranes of different lipid compositions but with a distinct energetic response. In addition, a more complex event was observed in the presence of lipid rafts. In general, the isothermal titration is performed by several injections, but after each one the heat flux tends to return to the equilibrium that is reflected in the return to the baseline level. In the case of DRMs, the return to the baseline level failed probably because of the presence of a very slow additional endothermic event. As shown in Fig. 5D and E, the Ebola fusion peptide was more efficient to induce aggregation of DRMs than vesicles of other lipid compositions (Fig. 5). However, the energetic response for the interaction between EBO_16_-W8A and DRMs from BHK-21 cells showed a small endothermic and exothermic contribution (Fig. 6G, a). In contrast, wtEBO_16_ induced an exothermic curve with a positive slope increasing with time after its interaction with DRMs (Fig. 6G, b). In both cases, the data show endothermic peaks as observed for other vesicles, although it has not been possible to discriminate the end of the endothermic process. For DRMs from Vero cells, the presence of sharp endothermic peaks followed by a broader exothermic peak was observed (Fig. 6I, a and b; Fig. 6J).

## Discussion

Although studies of the fusion domains of Ebola virus and Ebola pseudovirus have been at the forefront of research on cell entry in the filovirus infection cycle, many questions involving GP2 binding and membrane fusion remain unsolved. In this study, we showed that the Ebola fusion peptide is able to use lipid rafts as a target for virus entry into cells, which could explain why cholesterol-depleted cells have impaired Ebola virus GP-pseudotype-virion entry and fusion [35]. We demonstrated this ability in experiments of raft aggregation and cell-cell fusion (Fig. 1 and Fig. 5). Our results also demonstrate that Trp8 has an important role in the virus infection cycle. This was observed after substitution of this residue for alanine in the Ebola fusion peptide. The mutation abolished the ability of the peptide to acquire secondary structure in the presence of SDS micelles, as shown by Freitas et al. [12], suggesting that aromatic-aromatic interactions established by Trp8 and Phe12 are important in stabilizing a helical structure in the peptide [12]. Mutation at the same position affects the GP transport at the cell surface and its incorporation into VSV-Ebola virus pseudotype, in addition to creating a reduction in infectivity [11]. Furthermore, a structural change was directly correlated with the fusion activity (Fig. 3). In general, the Ebola fusion peptide can adopt more than one structure in an environmentally dependent manner. As it is frequently observed for peptides, the Ebola fusion peptide adopts a random conformation in solution [12], [14]. However, two other states have been described for the membrane-bound peptide: α -helical and β structures. The α-helix is observed in the interaction with membranes in the absence of Ca^2+^. This structure appears to be related to the ability of the peptide to cause membrane destabilization but not membrane fusion [13], [14], [36]. In fact, by DLS we observed that wtEBO_16_ is able to induce liposome aggregation in the absence of Ca^2+^, and although membrane fusion was not observed, the aggregation can be counted as an important step in this process (Fig. 5). In the presence of Ca^2+^, the peptide acquires a β-strand conformation by interaction with membranes that is related to the ability of this peptide to induce fusion [13], [14], [36]. Nevertheless, we suggest that the acquisition of a β-structure is not the unique feature needed for fusion since wtEBO_16_ was not able to induce fusion of PC∶PE∶SPM∶Cho liposomes, in spite of the high β-structure content. Thus, the correlation between structure and fusion cannot be a simple general rule.

**Figure 7:**
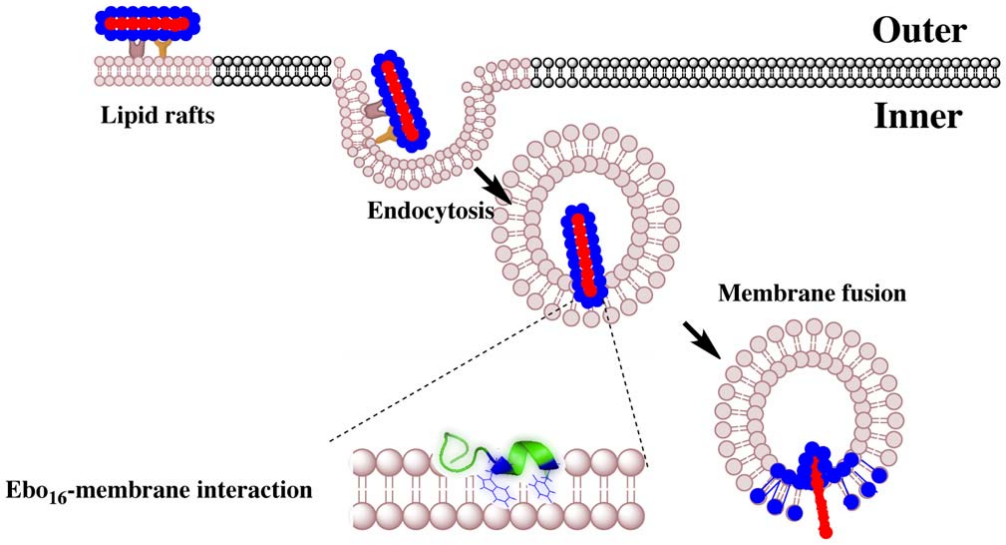
Schematic view of Ebola virus entry and fusion promoted by the fusion domain (EBO_16_). The interaction between Trp8 and Phe12 is emphasized with the tridimensional structure of EBO_16_ immersed in the lipid membrane (from PDB entry 2RLJ, [12]).

Figure 7 shows a schematic representation of the role of the Ebola fusion domain (EBO_16_) in the fusion process. The magnitude of the forces of the interaction between the Ebola fusion peptide and isolated microdomains (DRMs) from VERO cells was also highly striking. This sharp interaction decreased by about 10-fold when the membrane was saturated with free peptide. Furthermore, calorimetric results showed that the interaction between the Ebola fusion peptide and lipid rafts is complex, suggesting multiple energetic contributions (Fig. 6). The peptide conformational changes, lipid bilayer order/disorder and vesicle aggregation represent possible energetic contributions for the calorimetric profile. However, the understanding of the many contributions to the peptide/membrane interaction can give important insights into the filovirus infection cycle. In conclusion, we present a clear-cut demonstration of the ability of the Ebola fusion peptide to interact with lipid rafts (Figure 7), an interaction which is likely the crucial step in cell entry and the infection cycle of filoviruses.

## Materials and Methods

### Peptide

The Ebola fusion domain was purchased from Genemed Synthesis (South San Francisco, CA). Its purity and molecular mass were assessed by electrospray mass spectrometry, high-performance liquid chromatography and amino acid analysis. The purity was greater than 95%. Stock solutions were prepared by suspending the peptide at a final concentration of 1 or 2 mM in double-distilled water, where it is fully insoluble, and solubilization was achieved after addition of membrane models or dimethyl sulfoxide (DMSO). The peptide concentration was calculated from the absorbance at 280 nm using a molar absorption coefficient ε_280_ of 6,970 M^−1^cm^−1^.

### Vesicle size and aggregation

Vesicle sizes were characterized by DLS measurements in a Zetasizer Nano ZS (Malvern, UK). The measurement range was from 0.6 nm to 6 mm, and the analyzed range was from 50 to 2000 nm. The peptide concentration was 100 µM, and the liposome concentration was 1 mM. All solutions were filtered through 0.2 mm pore diameter membranes (Avanti Lipids Polar, Alabama, USA) into dust-free cells. The determination of the apparent hydrodynamic radius was performed using the CONTIN method [37].

### Fourier Transform Infrared

The FT-IR spectra were collected on a Nicolet Magna-IR 760 Fourier transform instrument (Nicolet, Madison, WI, USA). The spectral analysis was performed with the GRAMS 8.0 software (Thermo Scientific, USA). All spectra were collected in the presence of D_2_O.

### Cholesterol quantification

Following treatment with the Methyl-β-cyclodextrin (MβCD), Vero (Monkey cells), BHK-21 (Hamster cells) and C6/36 (Insect cells) monolayers were washed twice with PBS and treated with dissociation buffer-free enzyme (Gibco, CO) for a few seconds. Then, cells were scraped into PBS and centrifuged for 2 min at 730×g. The pellet was treated with the amplex red cholesterol assay kit (Molecular Probes, Invitrogen). The cholesterol was quantified by excitation at 530–560 nm and emission at 580–620 nm.

### Cell viability

The Vero, BHK-21 and C6/36 monolayers (ATCC) were washed twice with PBS and incubated in the presence of the MβCD and MTT. Four hours later the cells were incubated overnight in solubilization buffer (20% SDS, 50% dimethylformamide, pH 4.5).

### Lipid raft purification

All procedures were carried out on ice. Four T-150 and two T-75 glasses of cells were washed twice with phosphate buffer and treated for 10 sec with a dissociation buffer (enzyme-free). Cells were harvested and washed by centrifugation at 730×g for 2 min at 4°C in phosphate buffer and then once in phosphate buffer containing protease inhibitor cocktail (SIGMA, Saint Louis, MO, USA), 20 mM sodium orthovanadate activated (33), 2 mM aminoethyl-benzene sulfonyl fluoride (PMSF) and 1% triton X-100. The cells were then lysed by passage through a 28.7 needle 10 times. An equal volume of 80% sucrose was added to the mixer with the lysed cells and placed in the bottom of a sucrose gradient (40–5%) and centrifuged at 30,000 rpm for 24 h at 4°C using a SW40 Ti rotor. The gradient was fractionated, and the raft fraction was confirmed by dot blotting using cholera toxin B subunit-peroxidase conjugate (SIGMA, Saint Louis, MO, USA).

### Isothermal titration calorimetry

All measurements were carried out at 37°C in a VP-ITC from MicroCal, Llc. (Northampton, MA, USA). All solutions were degassed for 5 min prior to use. For each injection, 5 µL of a 20 mM stock solution of LUVs was injected into the sample cell (V = 1.422 mL) containing 100 µM of wtEBO_16_ or EBO_16_-W8A. Data handling (subtraction of baselines and heats of dilution, as well as peak integration) was performed with the Origin 7.0 software provided by MicroCal.

### Single molecule force spectroscopy

Force spectroscopy measurements were carried out at room temperature using a Nanowizard AFM (JPK Instruments, Germany). The AFM was mounted with a Nanoworld cantilever, and the constant spring was calibrated at each assay by the thermal noise method [38]. The Ebola fusion peptide was immobilized over an AFM functionalized tip. The AFM silicon tip was functionalized with carboxylic groups using radio frequency (RF) plasma treatment by applying acrylic acid (AA) vapor at 100 W plasma discharge for 5 min [39]. Afterwards, the AFM COO^−^ tip was covered with 10 µL of Ebola fusion peptide for 5 min and then washed with PBS. The microdomain vesicle was immobilized over slide glass. During the force-curve cycle the Ebola fusion peptide was allowed to interact with the microdomain. The unbinding force between Ebola fusion peptide and the microdomain was measured when the AFM cantilever was retracted. All experiments were performed under PBS buffer. The pulling speed of 2 mm/s was kept constant during all force-curve experiments. The force-distance curves were analyzed and plotted in a histogram of unbinding events to determine the probable unbinding force. Forces were plotted as a function of time before and after saturation with 1 mM wtEBO16.

## References

[1] Aman MJ, Bosio CM, Panchal RG, Burnett JC, Schmaljohn A (2003). Molecular mechanisms of filovirus cellular trafficking.. Microbes and Infection.

[2] Neumann G, Feldmann H, Watanabe S, Lukashevich I, Kawaoka Y (2002). Reverse genetics demonstrates that proteolytic processing of the Ebola virus glycoprotein is not essential for replication in cell culture.. Journal of Virology.

[3] Wool-Lewis RJ, Bates P (1999). Endoproteolytic processing of the Ebola virus envelope glycoprotein: Cleavage is not required for function.. Journal of Virology.

[4] Volchkov VE, Feldmann H, Volchkova VA, Klenk HD (1998). Processing of the Ebola virus glycoprotein by the proprotein convertase furin.. Proceedings of the National Academy of Sciences of the United States of America.

[5] Volchkov VE, Volchkova VA, Stroher U, Becker S, Dolnik O (2000). Proteolytic processing of Marburg virus glycoprotein.. Virology.

[6] Lee JE, Saphire EO (2009). Ebolavirus glycoprotein structure and mechanism of entry.. Future Virology.

[7] Stein BS, Gowda SD, Lifson JD, Penhallow RC, Bensch KG (1987). Ph-Independent Hiv Entry into Cd4-Positive T-Cells Via Virus Envelope Fusion to the Plasma-Membrane.. Cell.

[8] White J, Matlin K, Helenius A (1981). Cell-Fusion by Semliki Forest, Influenza, and Vesicular Stomatitis Viruses.. Journal of Cell Biology.

[9] Kielian M, Rey FA (2006). Virus membrane-fusion proteins: more than one way to make a hairpin.. Nature Reviews Microbiology.

[10] Tamm LK, Han X, Li Y, Lai AL (2002). Structure and function of membrane fusion peptides.. Biopolymers.

[11] Ito H, Watanabe S, Sanchez A, Whitt MA, Kawaoka Y (1999). Mutational analysis of the putative fusion domain of Ebola virus glycoprotein.. Journal of Virology.

[12] Freitas MS, Gaspar LP, Lorenzoni M, Almeida FCL, Tinoco LW (2007). Structure of the Ebola fusion peptide in a membrane-mimetic environment and the interaction with lipid rafts.. Journal of Biological Chemistry.

[13] Ruiz-Arguello MB, Goni FM, Pereira FB, Nieva JL (1998). Phosphatidylinositol-dependent membrane fusion induced by a putative fusogenic sequence of Ebola virus.. Journal of Virology.

[14] Suarez T, Gomara MJ, Goni FM, Mingarro I, Muga A (2003). Calcium-dependent conformational changes of membrane-bound Ebola fusion peptide drive vesicle fusion.. Febs Letters.

[15] Biswas S, Yin SR, Blank PS, Zimmerberg J (2008). Cholesterol promotes hemifusion and pore widening in membrane fusion induced by influenza hemagglutinin.. Journal of General Physiology.

[16] Alonso MA, Millan J (2001). The role of lipid rafts in signalling and membrane trafficking in T lymphocytes.. Journal of Cell Science.

[17] Simons K, Toomre D (2000). Lipid rafts and signal transduction.. Nature Reviews Molecular Cell Biology.

[18] Campbell SM, Crowe SM, Mak J (2001). Lipid rafts and HIV-1: from viral entry to assembly of progeny virions.. Journal of Clinical Virology.

[19] Stang E, Kartenbeck J, Parton RG (1997). Major histocompatibility complex class I molecules mediate association of SV40 with caveolae.. Molecular Biology of the Cell.

[20] Chan SY, Empig CJ, Welte FJ, Speck RF, Schmaljohn A (2001). Folate receptor-alpha is a cofactor for cellular entry by Marburg and Ebola viruses.. Cell.

[21] Nichols BJ, Kenworthy AK, Polishchuk RS, Lodge R, Roberts TH (2001). Rapid cycling of lipid raft markers between the cell surface and Golgi complex.. Journal of Cell Biology.

[22] Simmons G, Rennekamp AJ, Chai N, Vandenberghe LH, Riley JL (2003). Folate receptor alpha and caveolae are not required for Ebola virus glycoprotein-mediated viral infection.. Journal of Virology.

[23] Volynsky PE, Polyansky AA, Simakov NA, Arseniev AS, Efremov RG (2005). Effect of lipid composition on the “membrane response” induced by a fusion peptide.. Biochemistry.

[24] Maia LF, Soares MR, Valente AP, Almeida FCL, Oliveira AC (2006). Structure of a membrane-binding domain from a non-enveloped animal virus - Insights into the mechanism of membrane permeability and cellular entry.. Journal of Biological Chemistry.

[25] Empig CJ, Goldsmith MA (2002). Association of the caveola vesicular system with cellular entry by filoviruses.. Journal of Virology.

[26] Anderson HA, Chen YZ, Norkin LC (1996). Bound simian virus 40 translocates to caveolin-enriched membrane domains, and its entry is inhibited by drugs that selectively disrupt caveolae.. Molecular Biology of the Cell.

[27] Werling D, Hope JC, Chaplin P, Collins RA, Taylor G (1999). Involvement of caveolae in the uptake of respiratory syncytial virus antigen by dendritic cells.. Journal of Leukocyte Biology.

[28] Wool-Lewis RJ, Bates P (1998). Characterization of Ebola virus entry by using pseudotyped viruses: Identification of receptor-deficient cell lines.. Journal of Virology.

[29] Ohtani Y, Irie T, Uekama K, Fukunaga K, Pitha J (1989). Differential-Effects of Alpha-Cyclodextrins, Beta-Cyclodextrins and Gamma-Cyclodextrins on Human-Erythrocytes.. European Journal of Biochemistry.

[30] Yancey PG, Rodriqueza WV, Kilsdonk EPC, Stoudt GW, Johnson WJ (1996). Cellular cholesterol effect mediated by cyclodextrins - Demonstration of kinetic pools and mechanism of efflux.. Journal of Biological Chemistry.

[31] Gallaher WR (1996). Similar structural models of the transmembrane proteins of Ebola and avian sarcoma viruses.. Cell.

[32] Takada A, Robison C, Goto H, Sanchez A, Murti KG (1997). A system for functional analysis of Ebola virus glycoprotein.. Proc Natl Acad Sci U S A.

[33] Bar S, Takada A, Kawaoka Y, Alizon M (2006). Detection of cell-cell fusion mediated by Ebola virus glycoproteins.. Journal of Virology.

[34] Schornberg K, Matsuyama S, Kabsch K, Delos S, Bouton A (2006). Role of endosomal cathepsins in entry mediated by the Ebola virus glycoprotein.. Journal of Virology.

[35] Yonezawa A, Cavrois M, Greene WC (2005). Studies of Ebola virus glycoprotein-mediated entry and fusion by using pseudotyped human immunodeficiency virus type 1 virions: Involvement of cytoskeletal proteins and enhancement by tumor necrosis factor alpha.. Journal of Virology.

[36] Gomara MJ, Mora P, Mingarro I, Nieva JL (2004). Roles of a conserved proline in the internal fusion peptide of Ebola glycoprotein.. Febs Letters.

[37] Ostrowsky N, Sornette D, Parker P, Pike ER (1981). Exponential Sampling Method for Light-Scattering Polydispersity Analysis.. Optica Acta.

[38] Hutter JL, Bechhoefer J (1993). Calibration of Atomic-Force Microscope Tips.. Review of Scientific Instruments.

[39] Vilani C, Weibel DE, Zamora RRM, Habert AC, Achete CA (2007). Study of the influence of the acrylic acid plasma parameters on silicon and polyurethane substrates using XPS and AFM.. Applied Surface Science.

