# Correction: Measuring the Strength of Interaction between the Ebola Fusion Peptide and Lipid Rafts: Implications for Membrane Fusion and Virus Infection

**DOI:** 10.1371/annotation/6a5deee5-b766-49e5-a4ef-6079c2b25830

**Published:** 2011-05-12

**Authors:** Mônica S. Freitas, Cristian Follmer, Lilian T. Costa, Cecília Vilani, M. Lucia Bianconi, Carlos Alberto Achete, Jerson L. Silva

The importance played by lipid rafts during the entrance of Ebola and Marburg viruses into the cells was previously proposed by Bavari et al. in 2002. In this article, the authors show that this behavior is linked to the membrane fusion step. Therefore, there should be references to the following papers in this article:

Bavari S, Bosio CM, Wiegand E, Ruthel G, Will AB, Geisbert TW, Hevey M, Schmaljohn C, Schmaljohn A, Aman MJ (2002). Lipid raft microdomains: a gateway for compartmentalized trafficking of Ebola and Marburg viruses. J Exp Med 195:593-602.

Panchal RG, Ruthel G, Kenny TA, Kallstrom GH, Lane D, Badie SS, Li L, Bavari S, Aman MJ (2003). In vivo oligomerization and raft localization of Ebola virus protein VP40 during vesicular budding. Proc Natl Acad Sci U S A 100: 15936-15941.

